# Scoping Review of the Driving Behaviour of and Driver Training Programs for People on the Autism Spectrum

**DOI:** 10.1155/2018/6842306

**Published:** 2018-08-28

**Authors:** Nathan J. Wilson, Hoe C. Lee, Sharmila Vaz, Priscilla Vindin, Reinie Cordier

**Affiliations:** ^1^School of Nursing and Midwifery, Western Sydney University, Hawkesbury Campus, Locked Bag 3, Richmond, NSW 2753, Australia; ^2^School of Occupational Therapy Social Work and Speech Pathology, Faculty of Health Sciences, Curtin University, GPO Box U1987, Perth, WA 6845, Australia

## Abstract

Gaining a driver's licence represents increased independence and can lead to improved quality of life for individuals and their families. Learning to drive a motor vehicle and maintaining safe on-road skills are often more difficult for people on the autism spectrum. Many countries currently have no autism-specific licencing requirements for learner drivers, and there is a general lack of ASD-specific support and training packages for individuals, their families, and driving instructors. This review synthesises the peer-reviewed literature about the driving characteristics of drivers on the spectrum and driver training available for the cohort. The evidence in this review showed that individuals on the autism spectrum drive differently from their neurotypical counterparts. There are shortcomings in tactical skills of drivers on the autism spectrum, but the extent to which this affects their own safety or the safety of other road users is unclear. Tactical skills can be improved through training programs. There are few autism spectrum-specific learner training programs available. Development of an effective training program will benefit individuals on the spectrum to learn to drive, be independent, and be safe on the road.

## 1. Introduction

People on the autism spectrum experience problems with social and communication skills that are associated with executive function deficits affecting working memory, motor coordination, attention, planning, mental flexibility, and visual perception [1, 2]. This means that learning to drive a motor vehicle and maintaining safe on-road skills are often more difficult for people on the autism spectrum. Many people on the autism spectrum face barriers to community inclusion, which can create lifelong obstacles to experiencing individual independence. In most countries, gaining a driver's licence represents increased independence and can lead to improved quality of life for individuals and their families [3]. In fact, a recent retrospective longitudinal study showed that fewer people on the autism spectrum had a licence and generally, they obtained their licence later when compared to their peers [4]. Although recent estimates suggest that prevalences of people on the autism spectrum in Western countries range from 0.6% to 2.2% [5–7]; as far as we are aware, in the Australian context, there are currently no evidence-based autism-specific driving interventions available to people on the spectrum to help counter community mobility limitations that affect social and community participation. This review synthesises the peer-reviewed literature about the driving characteristics of drivers on the spectrum and driver training available for the cohort.

Driving plays a critical developmental role during the transition to adulthood and is associated with greater chances of securing paid employment, accessing education, training and services, and maintaining social relationships [8, 9]. Learner drivers on the autism spectrum demonstrate problems with driving skill acquisition and performance and typically require more, and longer, driving lessons and need to take the practical driving test more often when compared to their neurotypical peers [2, 3]. Further, research shows that people on the autism spectrum are also at greater risk of being involved in motor vehicle accidents, which poses risks not only for them but also for other road users in the community [10, 11].

Driving requires higher-order executive function mechanisms to work in a coordinated fashion to respond to, manage, and cope with multiple demands and unexpected and unpredictable driving situations [10]. Research has shown that people on the autism spectrum may experience executive function deficits [2, 9], which will directly impact on their ability to develop driving skills and drive safely. Research investigating the driving behaviour of people on the autism spectrum found that they notice driving hazards but they displayed a delay in response to social hazards that required interactions with other road users, such as failing to give way to pedestrians who show clear intent to cross the road [12–14]. This is thought to be related to a combination of reduced processing of social stimuli and attention deficits, which is very problematic when learning to drive a motor vehicle—a process which requires collaborative problem-solving skills [3]. These challenges are further confounded by research supporting the notion that people on the autism spectrum have atypical eye-gaze patterns, such as prolonged fixation of the speedometer to check the car speed and scanning repeatedly on traffic-irrelevant roadside objects such as advertisement billboards [2].

Three reviews of the literature covering various aspects of driving and use of public transportation in people on the autism spectrum have been published. The first review by Classen and Monahan [15] presented an overview of evidence-based intervention and predictor studies of driving performance in young people with attention deficit hyperactivity disorder (ADHD) or autism spectrum disorder (ASD). A total of ten papers, published between 1995 and 2011, met the criteria for inclusion in this review with nine of the ten papers focussing on driving in people with ADHD. Only one observational case-control study included ASD participants and focussed on hazard recognition in young people with and without ASD, using a virtual reality video display interface [14]. Thus, this first review highlighted the paucity of studies in the area and the urgent need for more empirical studies. The second review focussed on barriers and facilitators associated with driving and public transportation in people with ASD [9]. The review included 14 studies and narratively summarised findings related to (1) difficulties faced in obtaining a driver's licence (e.g., handling unexpected changes, sustaining attention for long drives, merging into traffic, and limited ability to read facial expressions and gestures), (2) driver confidence (e.g., due to lower reaction time and anxiety), (3) driving behaviours (e.g., avoiding heavy traffic or highways or driving at night, adherence to speed regulation, and lane maintenance), and (4) strategies advocated to improve driving skills in ASD (e.g., direct communication, minimal verbal correction, and-short duration lessons; introduction to driving in low noise density/quiet areas; and strategies to address anxiety). A third review focussed on the likely effects that ASD has on driving abilities in young people [16]. The authors reported that drivers on the autism spectrum (1) were less likely to identify social hazards (i.e., males with ASD), (2) had increased reaction times, (3) had more tactical driving difficulties, (4) reported more traffic crashes, citations, and intentional driving violations, and (5) had poorer situational awareness skills than drivers without autism. A major implication of this review was the need for strategic and systematic approaches to train people on the autism spectrum to drive.

In summary, the body of knowledge on driving and ASD needs further consolidation. A comprehensive review of both empirical and grey literatures that focuses on barriers and enablers to obtaining a licence that are related to (a) people on the autism spectrum (skills, behaviours), (b) the learning environment (policy, driver-training practice, instructor, and physical/built environments), and (c) within-the-person-environment context is needed to guide the design of a training intervention on the person with autism spectrum disorder, as well as their driving instructors [1, 9]. Many countries currently have no autism-specific licencing requirements for learner drivers and there is a general lack of ASD-specific support and training packages for individuals, their families, and driving instructors [3, 4, 17].

## 2. Methodology

### 2.1. Aim

The aim of this scoping review was to synthesise the current peer-reviewed research literature about the driving behaviours of individuals on the autism spectrum and explore the available training programs for this cohort with a view to suggest future research that promotes better training outcomes for learner drivers on the autism spectrum.

### 2.2. Design

A scoping review of contemporary peer-reviewed research articles published in English between 2000 and August 2017 was undertaken. Seven databases were searched: PubMed, Scopus, ProQuest, Embase, Medline, CINAHL, and TRID.

### 2.3. Search Strategy

The following search terms were used as appropriate, for each database: “Child development disorders, pervasive” OR “child developmental disorder^∗^” OR “pervasive developmental disorder^∗^” OR autism OR Asperger OR “autism spectrum disorder^∗^” OR “autistic disorder^∗^” OR “Developmental disabil^∗^” AND “Automobile Driving” OR driving or “driv^∗^ training” OR “driver behaviour” OR “driver performance” OR “driver characteristic” OR “driv^∗^ education” OR “driv^∗^ testing” OR “driv^∗^ procedures” OR “driving hazard^∗^” OR “car driv^∗^”OR “automobile driv^∗^” OR “driv^∗^packages”.

### 2.4. Study Selection

**Figure 1:**
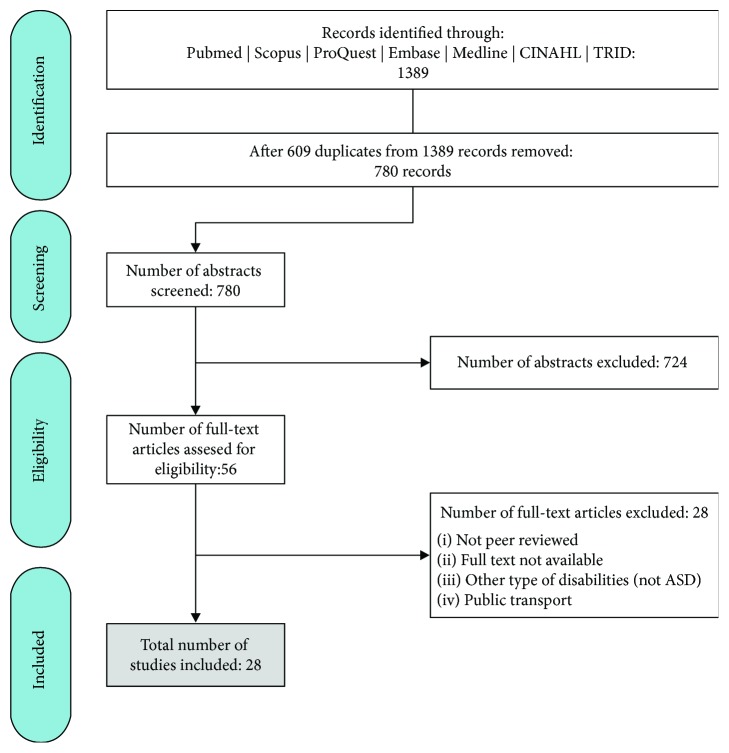
Flowchart of included studies.

The search yielded 1389 results from all seven databases. After removing 609 duplicates, a total of 780 articles were screened at an abstract level for inclusion. Studies were excluded if they did not involve people with ASD and if they did not focus on an aspect of driver training or driving skill development. After two authors reviewed the abstracts, 56 articles were deemed to be suitable for review at full-text level. Of the 56 studies reviewed at full-text level, 28 were excluded based on the following exclusion criteria: (1) not peer-reviewed article, (2) no full text available, (3) focus on other disabilities (not ASD), and (4) focus on transportation rather than independent driving. This resulted in a total of 28 studies for analysis; Figure 1 provides an illustration of the review process.

## 3. Results

All articles were grouped into four broad categories based on all author-agreed interpretation of each study's findings. These categories included (1) on-road driving behaviours and transport statistics reports on drivers on the spectrum (*n* = 7), (2) performance in driving simulators (*n* = 9), (3) performance in virtual reality driving (*n* = 9), and (4) barriers to obtaining a licence and training of drivers on the spectrum (*n* = 3). Although driving simulators represent a virtual reality context, the simulator category differs from the virtual reality driving category as the context of the latter was using devices such as a desktop computer, rather than a fully functional simulator. Between the two assessment regimes, the low-cost computer-based driving simulator has a better face validity in observing driving behaviours. Descriptive information was extracted from all articles and included the authors, year of publication, study design, objectives, population, and key findings. Study designs were described using the decision matrix recommended by the Centre for Evidence-Based Medicine [18]. This information is summarised in Table 1.

There was not a wide distribution of empirical studies from around the globe, with over 70% of the studies from the USA (*n* = 20), followed by Australia (*n* = 3), the UK (*n* = 3), Canada (*n* = 1), and Sweden (*n* = 1). Research on the topic area was mainly conducted in high-income countries. A total of 1481 participants on the autism spectrum were included across all 28 studies, with 79% of those participants being male. In terms of research designs, there were a total of 23 observational studies, two case studies, two review articles, and one driving simulator study with a quasi-experimental design. The published case study [19] used the same single male subject on the autism spectrum that was used in a comparison with a neurotypical male peer of the same age [20]. In the quasi-experimental study, the intervention group consisted of novice drivers on the autism spectrum (*n* = 51) and the comparison group (*n* = 333) was experienced licenced drivers without autism aged over 25 years [21].

## 4. Key Categories

### 4.1. On-Road Driving Behaviours and Transport Statistics Reports on Drivers on the Spectrum

Drivers on the autism spectrum were reported to have proportionally more traffic offences than their neurotypical counterparts (*p* < 0.05) [22]. This study also reported that drivers on the autism spectrum obtained their licence later than other drivers and self-rated their driving skills on a 10-point Likert scale as lower. Cox et al. [21] reported on a greater proportion of traffic offences in drivers on the autism spectrum. Other studies reported that drivers on the autism spectrum demonstrated decreased manoeuvring ability, particularly in left- and right-hand turns and an increased response time to traffic hazards, particularly in circumstances that required interaction with other road users (e.g., being hesitant to merge into another lane when other drivers had already gestured and reduced speed to allow the manoeuvre to happen) [14, 23]. Chee et al. [23] also found that drivers on the autism spectrum performed better than neurotypical drivers in rule-following aspects of driving, such as using the indicator and checking for traffic when approaching an intersection.

Performing complex driving functions that required multitasking skills (e.g., merging and using roundabouts) were often more difficult for the person on the autism spectrum [23, 24]. Driving tasks, or situations, that were sometimes difficult for drivers on the autism spectrum included driving in heavy traffic, night driving, maintaining the correct speed, lane maintenance, judging distance, and undertaking long journeys. In particular, drivers on the autism spectrum sometimes struggled to interpret the driving actions of other road users and found slight deviations from traffic rules of other drivers—a challenge and anxiety provoking [23].

### 4.2. Performance in Driving Simulators

All of the studies in this category used a comparison group of drivers not on the autism spectrum, except for one study by Monahan et al. [19]. Bishop et al. [12] and Brooks et al. [1] reported that in comparing drivers on the spectrum and the drivers in the control groups, there were no between-group differences in reaction time to hazard perception and motor response time during predriving assessments. In Brooks et al.'s [1] driving simulator study, when compared with the neurotypical controls, participants on the autism spectrum required an additional 30–35 minutes to complete 18 rounds of steering and pedal skill exercises.

Other driving simulator studies reported no between-group differences in (1) errors of maintenance of lane position and speed, (2) adjustment to distractions and poorer right-sided visual acuity [10], (3) response time in braking and overall driving ability [2], and (4) interpreting the gap between the front car, the speed, and the traffic flow of specific traffic scenarios [19]. Reimer et al. [25] also reported that participants on the autism spectrum showed different eye-gaze patterns. When responding to added cognitive demands, they positioned their vertical gaze higher and toward distant objects with more visual diversion. This can reduce the detection of hazards on the peripheral visual field of the individuals. The only quasi-experimental study included in the review used a driving simulator to training drivers on the spectrum [21]. The study showed that drivers on the autism spectrum had poorer baseline executive function and underperformed in tactical skills during simulated scenarios of unanticipated traffic demands [21]. Participants were trained in stages using a driving simulator program during one-hour training sessions across 10–12 weeks, focusing on alternated training of executive functions and tactical driving skills in driving. Outcome measures from the study showed that the training program significantly improved both executive function and driving skills.

### 4.3. Performance in Virtual Reality Driving

These studies all used some form of a desktop computer and screen as the basis of the virtual reality driving experience with driving-styled consoles (e.g., Logitech™ steering wheel) attached, to enable the participant to mimic driving. Six of the seven studies were led by various members from the same US Electrical Engineering and Computer Science research team. Many were proof of concept studies to determine whether the various data collection methods and tools can be augmented with a virtual reality program to detect between subject differences in areas, for example, such as eye gaze, EEG data, and physiological responses. The seventh study was from a team of UK researchers and reported that participants on the autism spectrum did not detect the time to arrival of two moving cars on a straight road as accurately as the comparison group [26]. Although this study used a virtual reality driving program, none of the participants were actual drivers and so the ability to draw real-life conclusions is limited.

### 4.4. Barriers to Obtaining a Licence and Training of Drivers on the Spectrum

Both drivers with ASD (learners and with a licence) experienced greater problems with driving and require more lessons and more tests than neurotypical drivers. Learning to drive was sometimes more difficult for the person on the autism spectrum as the process of reading, understanding, and converting driving theory into driving practice was demanding [3]. Unexpected changes to the usual routine or driving norms—where other drivers did not obey the traffic rules—were problematic for people on the autism spectrum [3]. Other reported problems included a lack of confidence, overconfidence, sensory overload, anxiety, poorer concentration, and greater distractibility.

Although all the research studies in this category were either observational or literature reviews that focussed on barriers, there were a few suggestions of potential ways to overcome these barriers. These included strategies to assist driving instructors, such as using direct communication and concrete instructions, providing shorter driving lessons, to commence each lesson in a quiet neighbourhood, and only giving the minimum amount of required information at a time [9]. There was a broad suggestion to use strategies to address anxiety; however, these would need to be individualised to the person on the autism spectrum. In Tyler's [17] four case studies with young adult males on the autism spectrum, some of these individualised strategies included using parent/learner/instructor communication logbooks to enhance consistency, structured language, tasks analysis, and breakdown, using a range of “what if” scenarios to increase insight into unpredictable driving and traffic situations and using visual markers to aid with judging distance.

## 5. Discussion

There is inconsistent evidence on the driving ability of people on the autism spectrum. For example, some studies reported that there is no difference between drivers on the autism spectrum and neurotypical counterparts in the maintenance of lane position and speed [8], response time in braking, and overall driving ability [2]. These studies were conducted either in simulated driving or in virtual reality environments generated by computer desktops. Conversely, when the ability of drivers was assessed in naturalistic on-road driving environments, drivers on the autism spectrum experience difficulty in complex traffic scenarios, especially driving tasks that required nonverbal communications with other drivers [23]. In addition, when compared with neurotypical controls, drivers on the autism spectrum had difficulty in multitasking and performing complex driving tasks and consistently maintaining a safe speed. They were also observed to have decreased manoeuvring ability and increased in response time to traffic hazards [23]. Unexpected changes to the usual driving routine or driving norms from other drivers distracted the attention of drivers on the autism spectrum and induced performance anxiety. When responding to added cognitive demands, drivers on the autism spectrum had poorer baseline executive function and performed worse on general and tactical driving [23].

Similarly, discrepancies were found by Cox et al. [21] who reported a greater proportion of traffic offences in drivers on the autism spectrum, whereas Chee et al. [23] found that drivers on the autism spectrum performed better than neurotypical drivers in rule-following aspects of driving. A possible explanation for these discrepancies is that the Cox et al. [21] study observed drivers on the autism spectrum using a driving simulator assessment, which is an artificial context. Consequentially, the real-life behaviour changes of drivers on the autism spectrum in responding to high-demand traffic scenarios were not known. This notion is supported in a validity study using driving simulators to assess driving behaviours of older drivers. Lee et al. [27] reported that only 67% of driving behaviours observed in a driving simulator was transferable to an on-road driving environment. Simulated driving environments might not be sensitive enough to detect changes of the driving performance. On-road driving is the gold standard to assess and observe ability of drivers. Drivers on the autism spectrum underperform in some aspects of driving or drive differently in naturalistic environments, but there is insufficient evidence to support the notion that drivers on the spectrum are more at risk of being involved in an accident than their neurotypical counterparts [28].

The observational studies [8, 26] using cross-sectional designs reported drivers on the autism spectrum to have difficulty in interpreting the driving actions of other road users. The eye-gaze patterns of drivers on the autism spectrum demonstrated an increased response time in detecting hazards, especially those associated with ineffective interpretation of the intentions of other drivers by means of body gesture and social clues. Insufficient attention to the traffic events detected on the peripheral visual fields can be hazardous and unsafe on road [26].

Individuals on the autism spectrum appear to have decreased ability to multitask and manoeuvre through complex traffic scenarios. To accomplish these tasks, drivers on the autism spectrum require training on the tactical level skills of the Michon's model of driving [29]. Tactical behaviour in driving involves striking a balance between the demands of driving and the driver's ability to drive safely in accordance to the road rules [30]. Successful acquisition of the tactical skills may allow drivers on the autism spectrum to reserve their cognitive capacity to cope with tasks with high number of executive functions and decision-making demands.

In identifying the training of the individuals on the spectrum, there is no experimental study aimed at testing novel ways to train individuals on the autism spectrum to obtain their driver's licence. In the current review, most observational studies described the barrier individuals on the autism spectrum experience in getting a driver's licence. None of them focussed on investigating the best strategies to help individuals on the spectrum to obtain their driver's licence.

### 5.1. Future Research

The current literature reviewed provides a preliminary driving profile of individuals on the autism spectrum in simulated contexts or during on-road assessments with predetermined routes. Given that individuals on the autism spectrum are susceptible to stress and anxiety, their driving experience is very likely to deteriorate in a test environment. For this particular cohort, research into the individuals in naturalistic driving environments will generate a more comprehensive driver profile. Driving is essential for individuals on the spectrum, but they showed a relatively low take-up rate of formal driving licence. There is limited research on how to effectively train individuals on the autism spectrum to drive independently and safely. Future research should focus on identifying strategies and the best practice of training to support individuals on the autism spectrum to get their licences.

### 5.2. Limitations

Most of the studies included in this review used to establish a profile of drivers on the spectrum were based on observations in driving simulator and/or virtual reality settings. This is a major limitation, given the low transferability of observations from a driving simulator environment to real-life on-road driving [27]. Findings from this scoping review should be interpreted with caution, as we only included studies published in English; grey literature, books, and theses were outside the scope of our review. Further, there are eight studies that employed self-report methodology to collect information from individuals on the autism spectrum on their ability to drive. Although there is no incentive for the participants to have falsely reported their driving ability, their reporting may have inflated their ability to drive and may have been influenced by recall bias.

## 6. Conclusion

Synthesis of the evidence in this review showed that individuals on the autism spectrum drive differently from their neurotypical counterparts. There are shortcomings in tactical skills of the drivers on the autism spectrum, but the extent to which this affects their own safety or the safety of other road users is not yet clear. Tactical skills can be improved through targeted training programs, specifically designed to accommodate the driving characteristics of the autism population. There are few autism spectrum-specific learner training programs available. Development of an effective training program will benefit the individuals on the spectrum to learn to drive, be independent, and be safe on the road.

## Figures and Tables

**Table 1:** 

Authors, year (country)	Type of study (study design)	Objective	Population	Key findings	Study limitations
*On-road driving behaviours and transport statistics reports on drivers on the spectrum (n* = 7)					
Chee et al. 2015 (Australia) [28]	Cross-sectional study	To understand people with ASD's viewpoints on driving using Q-methodology (e.g., whether or not they are confident and prefer driving than other transportation).	*n* = 100 50 with ASD (MA = 21.8 years; 82% male) 57 young adults without ASD (MA = 23.6 years; 72% male)	(i) Some young adults with ASD perceived themselves as confident and independent drivers. (ii) Some preferred to use public transportation or walk; anxiety was a barrier to driving for some. (iii) 34% with ASD had a driver licence compared to 68% of typical developing.	Diagnosis of ASD based on self-report Underrepresentation of nondrivers in the control group.

Chee et al. 2017 (Australia) [23]	Cross-sectional study	(i) To assess and compare the driving performance of drivers with and without ASD in suburban traffic environment (ii) To explore how the symptomatology of ASD hinders or facilitates the on-road driving performance	*n* = 37 16 with ASD (MA = 25.43 years; 86% male) 21 TD (MA = 27 years; 93% male)	(i) Drivers with ASD underperformed in vehicle manoeuvring, especially at left turns, right turns (hesitant and slower), and pedestrian crossings. (ii) Drivers with ASD outperformed the TD group in rule-following aspects such as using the indicator at roundabouts and checking for cross-traffic when approaching intersections.	Being small in sample size, the participants of this study may not be representative of the broader population and can impact on the generalisability of the results. Assessor bias—not blinded to diagnosis of participants or the purpose of the study No use of independent assessor or interrater reliability checks Impact of environmental factors, such as time of day and weather conditions on driving performance, cannot be disregarded. Driving route is developed by a researcher using the route of convenience for experimentation purposes; whether it mimics traffic demands on a normal road is questionable.

Cox et al. 2012 (USA) [24]	Cross-sectional study	An online survey to understand driving and ASD by surveying parents/caregivers of adolescents and young adults with ASD, who had driving licence or were in the process of learning to drive	*n* = 123 parents/caregivers (mother 81%, father 13%, and caregiver 6%) The mean age of their child with ASD was 19 years, 73% male; 83% Asperger's/high-function ASD, 9% PDD-NOS, 3% autistic disorder, and 5% others	(i) Parents were active in the teaching process (mother 81%; father 62%). (ii) 48% had a driver's licence, 29% possessed a learner's permit, and 70% of the parents stated that ASD affected driving ability. (iii) Particular challenges for people with ASD are complex driving demands (e.g., merging into traffic, traffic awareness, multitasking, handling unexpected changes, and sustaining attention on a long drive) (iv) Other difficulties include interpreting the actions of other drivers (e.g. reading their nonverbal social cues when in ambiguous situations).	Diagnosis of ASD is not confirmed through review of medical records Confounding impact of comorbidity is not accounted. Respondent bias (use of caregivers other than the person on the spectrum) limits validity. Use of a sample that was nonrepresentative; data collection is limited to internet survey.

Daly et al. 2014 (USA) [22]	Cross-sectional study	An online survey using a modified version of the Driver Behaviour Questionnaire to determine whether differences exist between adult drivers with ASD and non-ASD adult drivers in terms of (i) driving history and driving behaviours, (ii) the rates of reporting violations/mistakes/driving slips as defined by DBQ, and (iii) the relative risk of such behaviours as quantified by the DBQ.	*n* = 172 78 licenced drivers with ASD (MA = 32.9 years; 56% male, 98.7% autism/Asperger's, and 1.3% PDD-NOS) 94 non-ASD drivers (MA = 35.5 years; 31% male)	(i) Drivers with ASD obtained their driver's licence approximately 2 years later that non-ASD drivers. (ii) Drivers with ASD drove less days per week and had significantly lower ratings of their driving ability and a higher number of traffic violations. (iii) Drivers with ASD were also more likely to place voluntary restrictions on driving.	The pilot study relied on anonymous self-report. Participants, due to poor insight or difficulty in comparing their own driving behaviour with other drivers, may underreport their symptoms and overrate their driving ability. Report of driving behaviours through the use of standardized self-report measures would have improved the study rigor. Only the use of internet outlets for data collection may limit sample representativeness. Impact of comorbidity cannot be disregarded.

^∗^ Deka et al. 2016 (USA) [31]	Cross-sectional study	An online survey to learn about the travel patterns, needs, and barriers people with ASD encounter regarding the use of different transportation modes.	*n* = 703 Male : female = 3 : 16	(i) Only 9.3% of the adults with ASD had a driver's licence and many are using it as identity card rather than a licence to drive. (ii) 55.3% of the drivers with ASD (*n* = 47) had difficulty dealing with traffic, 34% mentioned difficulty caused by distractions near roads; 27.7% mentioned difficulty judging distance, and 27.7% had difficulty with parking. (iii) Because of these difficulties, 26.1% did not drive at all.	Low representation of views of ASD adults who had a driving licence and that have driven a car regularly

Huang et al. 2012 (USA) [32]	Cross-sectional study	An online survey to compare the characteristics of age-eligible driving and nondriving teens and explore the driving outcomes for teens with higher functioning autism spectrum disorders	*n* = 235 (MA = 16.39 years; 83%; 24.7% autistic disorder, 14.5% PDD-NOS, 1.5% ASD, 46.4% Asperger's, and 4.7% others)	(i) 63% of teens currently drive or plan to drive. (ii) 29% who are eligible to drive currently drive. (iii) Driving predictors included individualised education plans with driving goals, indicators of functional status, and parent experience with teaching teens to drive. (iv) 12% of teens received driving citations, and 12% of teens had been involved in a motor vehicle crash.	Respondent bias (use of caregivers other than the person on the spectrum) limits validity. Selection bias leading to nonrepresentative samples: families of teens who currently drive or plan to drive are more likely to participate in this study

Sheppard et al. 2017 (UK) [33]	Case control study	Videos of 20 different driving situations with inbuilt hazards to (i) compare the ability of identifying the driving hazards by stopping the video and pointing to the hazard between people with ASD and without ASD and (ii) define whether people with ASD have difficulty in responding to hazards or orienting to them.	*n* = 35 18 ASD males (MA = 18.79 years) 17 TD males (MA = 18.19 years)	(i) Although nonsignificant, participants with ASD showed a slower fixation on the hazard source and a slower orientation to the hazards. (ii) Greater attentional capture in the time preceding the hazards' onset was associated with lower verbal IQ. (iii), Suggesting individuals with ASD may distribute or direct their attention differently when identifying driving hazards.	The study is limited to young male ASD adults with no specific training or driving experience. Results cannot be generalised to ASD drivers with a licence or of different age ranges or females with ASD.

*Performance in driving simulators (n* = 9)					
Bishop et al. 2017 (USA) [12]	Cross-sectional study	To investigate how drivers with ASD respond to social and nonsocial hazards in a driving simulator compared to typically developing drivers using a driving simulator	*n* = 32 16 ASD drivers (MA = 23.88 years; 15% male) 16 TD drivers (MA = 22.94 years; 15% male)	(i) There was no difference in reaction time to social versus nonsocial hazards for drivers with ASD. (ii) Typically developing drivers reacted more quickly to social hazards as compared to nonsocial hazards.	Small sample size and underpowered Low scores on ASD symptomatology: ASD Social Responsive Scale. Unclear of any real-life translation of the simulated driving assessment

Brooks et al. 2016 (USA) [1]	Cross-sectional study	To investigate the utility of using a driving simulator and interactive exercises (steering and pedal tasks) to address the motor aspects of predriving skills of young adults with ASD	*n* = 41 10 ASD (MA = 15.9 years; 10 male) 31 TD (MA = 16.7 years; males = 18)	(i) Minimal performance differences were observed between the two groups in terms of the motor aspects of predriving skills. (ii) Participants with ASD needed more time (30–35 minutes) to complete the 18 interactive steering and pedal exercises. (iii) The interactive exercises and the process used worked well to address motor-related aspects of predriving skills in young adults with ASD.	Small sample size and underpowered Females are excluded in the ASD group. Unclear of any real-life translation: interactive exercises do not include any road scenes that help to eliminate the sensory overload, anxiety, and stress that participants might experience in naturalistic driving environments

Classen and Monahan 2013 (USA) [15]	Literature review	To conduct an evidence-based review of intervention studies and predictor studies related to driving outcomes in teens with ADHD or ASD	*n* = 10 intervention studies (2 RCT, 7 quasi-experiments, and 1 prospective observational study) Quasi-experimental studies: 7 on ADHD and 1 on ASD	(i) The ASD group perceives driving hazards via video clips but has difficulty in perceiving hazards if the task also requires processing of social information. (ii) The ASD group responds slower to hazard perceptions compared to controls. (iii) An instrumented vehicle, simulator training, and parent involvement invention had positive effect for speeding and hard braking for teens with ADHD. (iv) Male drivers with ADHD improved in hazard perception response times following training with hazard videos.	Heterogeneity among the studies: variability in age Inclusion criteria restricted to articles published in English and male participants Using different driving simulators, driving scenarios, outcome measures of assessment, failure to control for any prior rehabilitation, or clinical interventions lessened the methodological rigour of the studies. Grey literature is not included in the search.

Classen et al. 2013 (USA) [8]	Observational study	Using a driving simulator to examine the demographic, clinical, and simulated driving (type and number of driving errors) differences between teens with ADHD-ASD, healthy controls (HCs).	*n* = 44 22 ADHD-ASD (MA = 15.05 years; 17% male) 22 healthy controls (MA = 14.32 years; 59% male)	(i) Teens with ADHD-ASD performed more poorly on right eye visual acuity, selective attention, visual motor integration, cognition, and motor performance. (ii) Teens with ADHD-ASD made more errors on the driving simulator regarding visual scanning, speed regulation, lane maintenance, adjustment to stimuli, and total number of driving errors. (iii) Compared with HC teens, teens with ADHD-ASD may have more predriving skill deficits.	Sample of convenience: a Caucasian sample is not representative of the population of interest. Small sample size: underpowered study Selection bias: more concerned parents and teens with better insight enrolled in the study Berkson's bias: test taking and driving behaviours could be influenced by the evaluator's sitting next to the client. Hawthorne bias: the test-taking and driving behaviours are influenced by the testing site and social conditions. Did not control for medication effects on driving Simulator study: questionable of real-life driving equivalence

Cox et al. 2016 (USA) [2]	Cross-sectional study	To examine the relationship between driving performance and executive functioning for novice drivers, with and without ASD, using a driving simulator	*n* = 44 males 17 ASD (MA = 18.28 years) 27 healthy controls (MA = 16.59 years)	(i) ASD drivers had significantly slower reaction times during steering but not braking. (ii) ASD drivers demonstrated impaired working memory functioning, such that adding working memory demands resulted in a significant decrement in their driving performance relative to control drivers. (iii) ASD drivers demonstrated poorer overall driving ability than the control drivers.	Simulator study—questionable real-life equivalence Diagnosis based on parent report: medical records/doctors not consulted Cognitive measure is not used to assess intellectual functioning. Equivalence of comparison groups questionable: the control group was younger licenced drivers, whereas the ASD group had learner's permits. Previous driving experience was not accounted for in both groups.

Cox et al. 2017 (USA) [21]	Pre- and post-quasi-experimental studies	Using a virtual reality driving simulator to investigate how novice drivers with ASD differ from experienced drivers and whether virtual reality driving simulation training (VRDST) improves ASD driving performance	51 novice ASD drivers (MA = 17.96 years; 78% male) 23 in routine training (RT) 14 in standard VRDST 14 in automated VRDST 18 in eye-tracking VRDST only 333 normative licenced comparison group (MA = 40 years; 73% male)	(i) ASD drivers showed worse baseline executive function (EF) and driving skills than experienced drivers. (ii) ASD drivers performed worse than normative drivers on general tactical driving (iii) Both standard and automated VRDST significantly improved driving (e.g., better steering and speed control) and EF performance compared to RT. (iv) Eye-tracking VRDST did not significantly improve tactical performance relative to RT.	No random assignment of subjects to groups Small sample size: the study can be under powered

Monahan et al. 2012 (USA) [19]	Single subject case study	To illustrate the predriving skills of a teen with ADHD/ASD using a driving simulator	1 male with ASD/ADHD, 15 years old	(i) Demonstrated significant impairments related to visual motor integration and attention shift (ii) Did not perform visual scanning for traffic at cross streets and during lane changes (iii) Approached all interactions with excessive speed (iv) Poor understanding of traffic flow (v) Made gap acceptance error related to being overcautious	Single case study design: inherently faces the lack of representativeness of general population

Monahan et al. 2013 (USA) [20]	Cross-sectional study	Using a driving simulator to compare the predriving skills of a teen with ADHD/ASD to an age- and gender-matched healthy control.	1 male with ADHD/ASD, 15 years old 1 youth without ADHD/ASD, 15 years old	(i) Youth with ADHD/ASD demonstrated impairments in the ability to shift attention, perform simple sequential tasks, integrate visual-motor responses, and coordinate motor responses (ii) Youth without ADHD/ASD demonstrated intact skills in these abilities. (iii) Youth with ADHD/ASD made 44 driving error, while the youth without ADHD/ASD made 17 during the driving. (iv) Youth with ADHD/ASD had more lane maintenance, visual scanning, and speeding errors compared to the youth without ADHD/ASD.	Single participant in each comparison group

Reimer et al. 2013 (USA) [25]	Cross-sectional study	Using a driving simulator to explore driving behaviour and visual attention in young adult drivers with high-function (HF) ASD in comparison with a community sample of nonaffected individuals matched for age and sex	*n* = 20 10 male with HF-ASD (MA = 20.2 years) 10 matched controls (MA = 20.7 years)	(i) Youth with HF-ASD performed comparable to controls in terms of the frequency of simulated crashes and vehicle control. (ii) Youth with HF-ASD displayed a nominally higher and unvaried heart rate compared to controls. (iii) Youth with HF-ASD showed a gaze pattern suggestive of a diversion of visual attention away from high-stimulus areas of the roadway when responding to increased cognitive demands (cell phone task; continuous performance task). (iv) Youth with HF-ASD tended to position their vertical gaze higher and away from nearer objects and more toward the distance than controls.	Sample of convenience—Caucasian comparison groups are not representative of the population of interest—limits generalisability of results. Small sample size: underpowered study Without screening on age and cognitive functions, a convenient sample from the community as the control group Assessment of intellectual functioning or ASD was not obtained. Degree to which outcome measures relate to other covariates such as driving frequency and exposure is not reported.

*Performance in virtual reality driving (n* = 9)					
Bian et al. 2016 (USA) [34]	Study one: descriptive study Study two: case control experimental design, random allocation of members to comparison groups	To assess the feasibility of outcome measures for and responsiveness of virtual reality driving package	4 teenagers with ASD—3 male and one female 2 in the performance-sensitive system (PS) group (degree of difficulty changed based on performance only) 2 in the engagement-sensitive system (ES) group (degree of difficulty changed based on performance and engagement)	(i) All participants reported that they “enjoyed the game” and noticed the changes in task difficulty. (ii) The physiological data was successfully used to assess the user's engagement level and performance enabling the dynamic adjustment of the driving difficulty level.	Small sample size: underpowered study Equivalence of groups was questionable: effects of neither age nor driving experience are sufficiently controlled for the studies Study design does not take into account the possibility of comorbidity of ADHD in the sample group. Unclear of any real-life translation of the driving simulator study.

Fan et al. 2018 (USA) [35]	Descriptive study	To generate proof of concept data to determine if EEG data is useful in ASD driving interventions	20 ASD (MA = 15.29 years; 19 males and 1 female)	(i) EEG-based group level classification models are feasible for recognizing affect and workload recognition in individuals with ASD in the context of using a desktop virtual reality driving program. (ii) Although promising, the applicability of this approach to collecting outcome data in other interventions requires further development.	Small sample size of the neurotypical comparison group Limits with interpreting EEG data of virtual reality environments as in real-life contexts

Sheppard et al. 2010 (UK) [14]	Cross-sectional study	To compare the ability of identifying 10 different driving hazards by stopping the video and pointing to the hazard between people with ASD and without ASD	*n* = 44 23 males with ASD (MA = 18.55 years; 30% ASD and 70% Asperger's) 21 males without ASD (MA = 18.83 years)	(i) Participants with ASD identified fewer social hazards (those that involved people like pedestrians, cyclists), but not nonsocial hazards (e.g., cars). (ii) Participants with ASD were slower to respond than those without ASD.	Participants of the study are likely to have strategies prior to the experience to identify and respond to both types of hazard. Limited to only male participants Results cannot be generalised to ASD drivers with a licence or of different age ranges or females with ASD.

Sheppard et al. 2016 (UK) [26]	Cross-sectional study	To investigate whether individuals with ASD have difficulty judging the location of moving objects in a driving context using a time-to-arrival task on a desktop virtual reality program	*n* = 44 23 males with ASD (MA = 18.55 years; 7 with Autism and 16 with Asperger's) 21 males in comparison group (MA = 18.83 years)	(i) Participants with ASD were less accurate at predicting which of the two cars in the virtual reality program would arrive first at an intersection on a straight road. (ii) There were no differences between participants with ASD and the comparison group when the simulation was along a curved road.	Participants were all nondrivers so generalisability to drivers is not possible. Unclear of any real-life translation

Sheppard et al. 2017 (UK) [33]	Case control study	Videos of 20 different driving situations with inbuilt hazards to (i) compare the ability of identifying the driving hazards by stopping the video and pointing to the hazard between people with ASD and without ASD and (ii) define whether people with ASD have difficulty in responding to hazards or orienting to them.	*n* = 35 18 ASD males (MA = 18.79 years) 17 TD males (MA = 18.19 years)	(i) Although nonsignificant, participants with ASD showed a slower fixation on the hazard source and a slower orientation to the hazards. (ii) Greater attentional capture in the time preceding the hazards onset was associated with lower verbal IQ, suggesting that individuals with ASD may distribute or direct their attention differently when identifying driving hazards.	Limited to male young adults with ASD, but with no specific training or driving experience Results cannot be generalised to ASD drivers or of different age ranges or females with ASD.

Wade et al. 2014 (USA) [36]	Cross-sectional study	To determine if a novel virtual reality driving environment can detect between-group differences	*n* = 8 4 males with ASD (MA = 16.87 years) 4 TD controls (MA = 15.34 years; 3 males and 1 female)	(i) The ASD group experiencing a higher number of simulated driving failures (ii) Participants with ASD had a vertically higher and horizontally right visual gaze. (iii) The ASD group had a significantly higher skin conduction level and skin conductance response rate suggesting that participants with ASD may have greater anxiety.	Small sample size: underpowered study Unclear of any real-life translation

Wade et al. 2015 (USA) [11]	Cross-sectional study	To compare the effects on driving performance and gaze patterns using the gaze-contingent system and the gaze-insensitive, performance-based system	*n* = 18 12 males with ASD in the gaze-contingent group (MA = 14.65 years) 6 in the performance-based group (MA = 15.93 years)	(i) The performance-based group showed a significantly higher mean vertical and right-sided gaze component when compared to the gaze-contingent group. (ii) The performance-based group showed a decrease in trial failures from pre- to post-post.	Small sample size: underpowered study. Equivalence of groups was questionable.

Zhang et al. 2015 (USA) [37]	Descriptive study	To evaluate the feasibility of combining psychophysiological data collection with performance based data using a virtual reality program	10 with ASD (age range = 13 to 17 years)	(i) The best accuracy to assess cognitive load was by combining eye gaze and EEG data in a hybrid data analysis model.	Small sample size: underpowered study Applicability to real-life situations was questionable.

Zhang et al. 2014 (USA) [38]	Descriptive study	To determine if performance data and affective data can be combined to predict the optimum driving difficulty level of a virtual reality program	7 with ASD (age range = 13 to 17 years)	(i) Combining performance and affective state data was better at predicting the difficulty level when compared to separating the data. (ii) The number of driving performance failures and the enjoyment level had a strong relationship with the difficulty level.	Small sample size: underpowered study

*Barriers to obtaining a licence and training on drivers of the spectrum (n* = 3)					
Almberg et al. 2017 (Sweden) [3]	Descriptive study	Questionnaires with open and closed questions to explore the facilitators or barriers in driving education experienced by individuals with ASD or ADHD who obtained a learner's permit, from the perspective of the learner drivers and their driving instructors	*n* = 33 14 male ADHD 19 ASD (MA = 20.7 years; 16 males and 3 females) 9 driving instructors (4 with ADHD learner drivers and 5 with ASD learner drivers)	(i) Participants with ASD required twice as many driving lessons and more on-road test than those with ADHD. (ii) Reading difficulties meant converting theory into practice was difficult. (iii) Adjusting to on-road situations that were unfamiliar of deviated from the “textbook” script was challenging. (iv) Paying attention to other road users and road signs was reported to be a problem. (v) Hazard perception and the ability to regulate the driving according to the traffic conditions were difficult for ASD participants.	Small sample size: underpowered study Use of nonvalidated outcome measures Did not consider comorbidity as a confounder Did not consider impact of prior familiarity and experience in working with people with ASD and ADHD in measuring outcomes

^∗^Lindsay 2017 (Canada) [9]	Systematic literature review	To systematically review the literature on factors (e.g., barriers and facilitators) affecting driving and motor vehicle transportation experiences of people with ASD	22 studies from eight databases with 2919 participants (mean age 17.3 years 364 individuals with ASD 2555 parents of youth with ASD	13 studies focused on factors affecting driving including the following: (i) Challenges in obtaining a licence (e.g., handling unexpected changes, sustaining attention for long drives, merging into traffic, and limited ability to read facial expressions and gestures) (ii) Driving confidence (not confident in reaction time, much resulted from experiences of anxiety) (iii) Driving behaviours (e.g., avoiding heavy traffic or highways, driving at night, adhering to speed regulation, and lane maintenance) (iv) Strategies to improving driving skills (e.g., direct communication, providing lessons in short intervals, starting off in quiet areas, not giving too much information or verbal correction, and address anxiety).	Research conducted on March 2015 Inclusion restricted to articles published in English Grey literature not included

Tyler 2013 (Australia) [17]	Case study	To examine the problems faced by supervisors and instructors during training and the strategies that can be implemented to decrease the risks associated for drivers with Asperger's syndrome	Four case studies on the following: (i) 20-year-old male with Asperger's/ADHD (ii) 26-year-old male, with Asperger's/anxiety/depression (iii) 20-year-old male with Asperger's/ADHD/ (iv) 18-year-old male with Asperger's/dyspraxia	(i) Problems included being easily distracted during driving, poor concentration, anxiety, and sensory overload (ii) Strategies included are as follows: (a) Communication book for parents/learners/instructors to reinforce the sequence of tasks using a consistent approach are provided. (b) Regular lessons were conducted. (c) The use of keywords and structured language in instructions for consistency reduced confusion and anxiety. (d) Reduce discussions on topic of interest to increase focus on the driving task. (e) Break tasks down and working through smaller components in sequence to reduce anxiety. (f) Instructors used “what if” scenarios to broaden participant's understanding of unpredictable drivers and pedestrians. (g) Instructors used visual markers for judgement of distance, crash avoidance space, and indicator distance rules.	Interpretation of results of the study dependent on the sensitivity and integrity of the investigator Case study design: inherently faces the lack of representativeness of general population Efforts undertaken to ensure trustworthiness, credibility, transferability, dependability, confirmability, and rigour of the study were not reported.

^∗^Study included the collection of both driving and public transport data, with sufficient data about driving to be included in this review.
